# Supplementary material for: From sequence to enzyme mechanism using multi-label machine learning
Source: BMC Bioinformatics. 2014 May 19;15:150. doi: 10.1186/1471-2105-15-150 (PMC4229970; doi:10.1186/1471-2105-15-150)
Supplement: Additional file 2 — Java code of ml2db. Additional file ml2db_code.tar.gz contains the Java source code to run the multi-label machine learning experiments and save the results to database. The code’s Javadoc is included. [file 1471-2105-15-150-S2.zip › additional file 2/ml2db/ml2db/doc/mulan/evaluation/measure/package-tree.html]

mulan.evaluation.measure Class Hierarchy


JavaScript is disabled on your browser.


- Overview
- Package
- Class
- Use
- Tree
- Deprecated
- Index
- Help

- Prev
- Next

- Frames
- No Frames

- All Classes

# Hierarchy For Package mulan.evaluation.measure

Package Hierarchies:

- All Packages

## Class Hierarchy

- java.lang.Object
  - mulan.evaluation.measure.InformationRetrievalMeasures
  - mulan.evaluation.measure.MeasureBase (implements mulan.evaluation.measure.Measure, java.io.Serializable)
    - mulan.evaluation.measure.BipartitionMeasureBase
      - mulan.evaluation.measure.ExampleBasedBipartitionMeasureBase
        - mulan.evaluation.measure.ExampleBasedAccuracy
        - mulan.evaluation.measure.ExampleBasedFMeasure
        - mulan.evaluation.measure.ExampleBasedPrecision
        - mulan.evaluation.measure.ExampleBasedRecall
        - mulan.evaluation.measure.ExampleBasedSpecificity
        - mulan.evaluation.measure.LossBasedBipartitionMeasureBase
          - mulan.evaluation.measure.HammingLoss
          - mulan.evaluation.measure.HierarchicalLoss
        - mulan.evaluation.measure.SubsetAccuracy
      - mulan.evaluation.measure.LabelBasedBipartitionMeasureBase
        - mulan.evaluation.measure.LabelBasedFMeasure
          - mulan.evaluation.measure.MacroFMeasure (implements mulan.evaluation.measure.MacroAverageMeasure)
          - mulan.evaluation.measure.MicroFMeasure
        - mulan.evaluation.measure.LabelBasedPrecision
          - mulan.evaluation.measure.MacroPrecision (implements mulan.evaluation.measure.MacroAverageMeasure)
          - mulan.evaluation.measure.MicroPrecision
        - mulan.evaluation.measure.LabelBasedRecall
          - mulan.evaluation.measure.MacroRecall (implements mulan.evaluation.measure.MacroAverageMeasure)
          - mulan.evaluation.measure.MicroRecall
        - mulan.evaluation.measure.LabelBasedSpecificity
          - mulan.evaluation.measure.MacroSpecificity (implements mulan.evaluation.measure.MacroAverageMeasure)
          - mulan.evaluation.measure.MicroSpecificity
    - mulan.evaluation.measure.ConfidenceMeasureBase
      - mulan.evaluation.measure.LabelBasedAUC
        - mulan.evaluation.measure.MacroAUC (implements mulan.evaluation.measure.MacroAverageMeasure)
        - mulan.evaluation.measure.MicroAUC
      - mulan.evaluation.measure.LabelBasedAveragePrecision
        - mulan.evaluation.measure.MeanAverageInterpolatedPrecision (implements mulan.evaluation.measure.MacroAverageMeasure)
          - mulan.evaluation.measure.GeometricMeanAverageInterpolatedPrecision
        - mulan.evaluation.measure.MeanAveragePrecision (implements mulan.evaluation.measure.MacroAverageMeasure)
          - mulan.evaluation.measure.GeometricMeanAveragePrecision
    - mulan.evaluation.measure.RankingMeasureBase
      - mulan.evaluation.measure.AveragePrecision
      - mulan.evaluation.measure.Coverage
      - mulan.evaluation.measure.LossBasedRankingMeasureBase
        - mulan.evaluation.measure.ErrorSetSize
        - mulan.evaluation.measure.IsError
        - mulan.evaluation.measure.OneError
        - mulan.evaluation.measure.RankingLoss

## Interface Hierarchy

- mulan.evaluation.measure.MacroAverageMeasure
- mulan.evaluation.measure.Measure

- Overview
- Package
- Class
- Use
- Tree
- Deprecated
- Index
- Help

- Prev
- Next

- Frames
- No Frames

- All Classes
